# 

**Published:** 1872-05

**Authors:** 


### BOOK NOTICES.

"Fireside Science: a Series of popular Scientific Essays on Subjects connected with Every-day Life," by JAMES R. NICHOLS, A. M., M. D., editor of the "Boston Journal of Chemistry." Published by Hurd and Houghton, New York. 284 pp. 12mo, $1.50. A book which can be profitably read at every fireside.

"Report of the last year's Operations of the Pennsylvania Hospital for the Insane at Philadelphia, under the Superintendence of Thomas M. Kirkbride, M. D." There is no institution of the kind in the United States which is managed with greater skill, ability, and success, justly attributed to the intelligence, tact, and ability of the physician in chief, Dr. Kirkbride.

"Questions of Quarantine: the Nature and Prevention of Communicable Zymotic Diseases," by ALFRED L. CARROLL, M. D., of New York; to which is appended Dr. Marsden's admirable plan, with diagram, for a cholera quarantine station; price fifty cents. Published by F. Leypoldt, 712 Broadway, New York. In view of the possible presence of cholera in this country during the coming summer, it is not extravagant to say that every educated physician owes it to himself to obtain a copy of this valuable monogram, especially as Professor W. Marsden of Quebec is one of the very foremost of the medical men of the New Dominion, and is second to none on this continent in his intimate, thorough, and extensive personal knowledge and accurate observation of facts in connection with this terrible disease.

"Dental Register," by Prof. TAFT of Cincinnati, merits the patronage of every dentist in the United States. It is $3 a year, in its 26th volume, and now, as heretofore, has been conducted with judgment and ability. It might be taken with incalculable advantage in every family of growing children.

"New York Juvenile Asylum: 19th Annual Report to the Legislature, and to the Common Council, A. R. Wetmore, President." This charity, or rather this beneficence, conducts its operations at 176th Street and Tenth Avenue; the house of reception is at 61 West 13th Street. This is for children from eight years and under to fourteen, who from poverty, abandonment, or crime, are sent by magistrates, parents, guardians, or friends, or come of their own accord, as either unfortunate, pilferers, bad, beggars, truants, or disobedient; more than a thousand are taken care of every year for a longer or shorter time, are taught habits of industry, cleanliness, virtue, obedience, and morality; as well as to read, write, work, and then are supplied with homes in reputable families at a distance from their city associations. The report gives many exceedingly interesting letters from these saved little ones after having been at their new homes for some time, abounding in expressions of gratitude and love toward those who were the agents of their deliverance from lives of destitution and crime. All honor be to the noble-hearted President of the institution, who for these many years has given his influence, and time, and care, and money to such a humane cause; nor should the able, skillful, and faithful services of the Superintendent, Dr. S. J. Brooks, nor of the motherly and assiduous matron, Mrs. Emma A. Apley, pass unrecognized. This charity is commended most heartily to the consideration of men and women who have hearts large enough to appreciate such a work. If any incentive is needed, apply to the Secretary, William C. Gilman, 61 West 13th Street, for the last Report of the New York Juvenile Asylum. The reading of the letters is a series of fairy tales, all true.

"Hubbs's New National Map, prepared from the Surveys of the War Department, and United States Coast Survey," guarantees great accuracy; it embraces the whole country from the Atlantic to the Pacific coast, and is admirably executed by F. Meyer & Co., showing all the railroads up to date, and all river courses. Price ten dollars. One ought to be hung up in every family room in the nation as the best possible means of impressing on the memory of the children the relative position and size of the different States of the Union, to be a life-long benefit and satisfaction.

"Centenary Address before the New York Hospital," by James William Beekman, to which is added Plain, Concise, Practical Remarks on the Treatment of Wounds and Fractures, with an Appendix on Camp and Military Hospitals, by John Jones, M. D., Professor of Surgery in Kings College, New York, in 1775, nearly a hundred years ago; the whole in a neat pamphlet of some sixty pages. Mr. Beekman's address begins with what happened a hundred years ago, bringing up the history of the New York Hospital to the present time. Those memories of the olden time, and reminiscences of the men of action then, how they stir up our imaginations, our respects, and our reverences. The address has an interest and a life beyond that found in any novel, especially as it is a reliable record, given in elegant and scholarly phrase. But this address has a practical bearing in making the wisdom of a hundred years ago the highest wisdom of to-day, teaching that a hut is a better place for a sick man than a splendid hospital, with all its costly appointments, Dr. Rush having taught that sick men recover sooner and better in sheds and shanties than in the most superb hospitals. We commend the address to all men of education and thought, as full of suggestiveness in many important directions.

"St. Paul in Rome, or the Teachings, Testimony, and Fellowship of the Great Apostle," 340 pp. 12mo, published by Carter Brothers, New York, $1.50, being a series of sermons with a most interesting introduction by I. R. McDuff, D. D. Every traveller who visits Rome will derive great benefit from reading this book; it will aid him greatly in visiting celebrated places, while every Christian will be fed and comforted in reading the sermons delivered by this celebrated divine in Rome during the year 1871. It may be added here that another book, Hare's "Walks in Rome," noticed in a previous number, published by Routledge and Son, 416 Broome Street, New York, will do more towards enabling a visitor to see everything of interest to advantage than any book of travel yet published. A lady correspondent, a New Yorker, writes from Rome of this volume, that it is priceless as an aid to seeing the great city; that it gives additional interest to everything she sees.

"Detection of Criminal Abortion," by ELY VAN DE WARKER, M. D., corresponding member of the Gynæcological Society of Boston, dedicated to Horatio R. Storer, M. D., LL. B., whose brave words against the "Crime of the Period" have done much good in that direction. It is a book which can be read to great advantage by all professions, and by the intelligent of all classes. Sent in paper cover for fifty cents.

"Journal of Psychological Medicine, being a Quarterly Review of Diseases of the Nervous System, Medical Jurisprudence, and Anthropology," edited by Wm. A. Hammond, Professor of Diseases of the Mind and Nervous System in the Bellevue Hospital Medical College; $5 a year, single copy $1.50. D. Appleton & Co., New York, and Trübner & Co., London, publishers. A standard publication of medical literature, original and selected. Persons requiring treatment in mental and nervous affections, consulting Dr. Hammond, will be in safe hands.

"Small-pox, and the Predisposing Conditions," by CARL BOTH, 25 cents, published by Alexander Moore, Boston. The chief points are that vaccination has not proved to be an absolutely reliable preventive against small-pox; that better means are needed; that salt is the most scientific and certain preventive; that organic acids are a temporary substitute for salt; that a person with properly balanced blood cannot take small-pox; that a proper use of salt will eradicate small-pox at once and forever. So he says! Mere assertions, all.

"Parasites, Animal and Vegetable, of the Skin and Hair," by B. JOY JEFFRIES, A. M., M. D. Alexander Moore, publisher, Boston, $1.00. A practical monogram both for the profession and the people.

"Vatican Council, its Inside View, being a Speech of Archbishop Kendrick, of St. Louis," edited by Leonard Woolsey Bacon, with notes and additional documents, 80 cents; by the American Tract Society, 150 Nassau Street, New York.

"Forty Years' Fight with the Drink Demon, being a History of the Temperance Reform, as I have seen it," by CHARLES JEWETT, M. D. A most instructive and useful book, just issued by the National Temperance Society, 58 Reade Street, New York. $1.50, illustrated.

"Sketches from Life, or Illustrations of the Influence of Christianity," third series, by the American Tract Society, $1.50. Full of short recitals of actual histories, and can be read with great profit by all, and every page is full of interest.

"Threescore Years and Beyond," by Rev. Dr. W. R. DEPUY, published by Carlton & Lanahan, New York, 512 pp. 8vo. This is a book mainly for old people, prepared with great care, industry, and judgment. It gives short and succinct histories of the lives and last scenes of the eminent dead, beginning with the Patriarchs and coming down to John Knox, Elizabeth Fry, and others. It is a book of exceeding interest, showing in what peace the Christians of all ages have died, looking to Jesus. It is a book of enduring comfort to the aged everywhere, whose hopes are in the Beyond.

